# Exploring the Relationships Between Resilience and News Monitoring with COVID Distress in Health Profession Students

**DOI:** 10.1007/s40596-021-01444-9

**Published:** 2021-04-29

**Authors:** Allison Yu, Michael Wilkes, Ana-Maria Iosif, Margaret Rea, Alice Fisher, Jeffrey Fine, Ross Perry, Elizabeth Rice, Karl Jandrey, Erin Griffin, Andres Sciolla

**Affiliations:** 1 University of California, Davis School of Medicine, Sacramento, CA USA; 2 University of California, Davis School of Nursing, Sacramento, CA USA; 3 University of California, Davis School of Veterinary Medicine, Davis, CA USA

**Keywords:** Resilience, News Monitoring, COVID-19, Pre-clinical Students

## Abstract

**Objective:**

Alarming rates of anxiety and burnout in pre-clinical health profession trainees are now challenged by additional COVID-19 stressors. This study explored COVID-related stressors among first-year medical, physician assistant, nurse practitioner, and veterinary medical students. The authors examined associations between resilience, news monitoring, and COVID stress.

**Methods:**

Students completed an online questionnaire that included the Brief Resilience Scale at their matriculation in August 2019. Survey results were linked to demographic information collected by all schools. A follow-up survey in May 2020 included original questions on COVID-19 stressors and news monitoring. Statistical analyses included descriptive statistics and multivariable linear regression models.

**Results:**

Across schools, 74% (266/360) provided consent for the 2019 survey, and 76% (201/264) responded to COVID-19 questions in the follow-up 2020 survey. Students were “extremely” or “very” concerned about family members getting infected (*n* = 71, 76% School of Medicine (SOM); *n* = 31, 76% School of Nursing (SON); *n* = 50, 75% School of Veterinary Medicine (SVM)) and curriculum schedule changes (*n* = 72, 78%, SOM; *n* = 28, 68% SON; *n* = 52, 79% SVM). Greater frequency of COVID news monitoring was associated with greater COVID-related stress (*p* = 0.02). Higher resilience at matriculation was associated with lower COVID-related stress ten months later (*p* < 0.001).

**Conclusions:**

Amid COVID-19 uncertainty, health science schools should address the immense student stress regarding curriculum disruptions. The results of this study underscore the powerful role of resilience in protecting against stress not only during the known academic rigor of health professions training but also during unprecedented crises.

In January of 2020, the first reported case of coronavirus disease 2019 (COVID-19) appeared in the USA; shortly after, the first community-spread case was treated in Sacramento, California [1]. Since then, healthcare workers have scrambled to treat COVID-19 patients and have often faced limited access to protective equipment, testing, and treatments. The psychological impact of COVID-19 has been studied in frontline healthcare workers, non-frontline healthcare workers, and the general public, with subsequent implementation of mental health protection programs [2–8]. Few studies have examined the effect of COVID-19 on the mental health of pre-clinical health science trainees [9].

Prior to the COVID-19 outbreak, a large body of evidence identified a high prevalence of stress, anxiety, and burnout among health professions students [10–16]. Stress and burnout progress from the pre-clinical years and predict decreased mastery of clinical tasks, more frequent medical errors, lower sense of responsibility to society, lower patient satisfaction, less frequent use of evidence-based practice, cynicism over patients with chronic illness, and poorer physical health following graduation [17–22]. Pre-clinical students now face compounded COVID-19-related stressors that distance them from their anticipated professions, including revised course structures, new online requirements, social isolation, and reduced student-patient and student-faculty interactions. To minimize the snowballing mental health concerns exacerbated by the current pandemic, it is essential to identify modifiable factors that may mitigate pre-clinical student stress. Resilience, often targeted as an interventional attribute due to its stress-protective effect, may be one such modifiable variable.

Resilience is broadly defined as a multidimensional set of attributes that allows us to “bounce back” and adapt in the presence of adversity [23]. There are many proposed attributes of resilience, including self-compassion, adaptive coping, self-efficacy, and mindfulness, all of which have a protective effect against burnout, anxiety, and depression [24–27]. Resilience is powerful in its dynamic nature and can be nurtured over time [28]. Previous studies have identified strategies for building resilience in healthcare workers such as seeking positive professional relationships, encouraging personal reflection, and achieving life balance [24, 29]. These strategies have been utilized in healthcare professionals to successfully combat stress from daily workplace challenges, such as high patient volume, low autonomy, and limited support from senior faculty [30–32]. However, no study has investigated the role of resilience in pre-clinical healthcare professionals during crises such as the COVID-19 pandemic.

One under-explored mediator of COVID-related stress is how frequently individuals monitor the news for updates pertaining to COVID [33, 34]. While accessible, up-to-date information about COVID spread and treatments was associated with lower stress, anxiety, and depression, the amount of time spent thinking about COVID, conversely, was associated with higher anxiety [4–6]. These findings appear to be at odds, as the act of seeking accurate COVID-related information presupposes thinking about COVID. It is also possible that processing COVID-related news may catalyze rather than mitigate compulsory COVID-related thoughts. This paradox may be answered by personality differences, media sources of news, and frequency of news monitoring.

From an interventional standpoint, the relationship between news monitoring and COVID-related stress is particularly intriguing because adjusting news monitoring frequency is a rapidly achievable goal. In light of progressive mental health concerns in health professions trainees beginning from matriculation, many schools have implemented wellness initiatives. Some initiatives do not gain traction with students because the proposed mindset and lifestyle changes do not feel tangible, accessible, or achievable. Additionally, a subset of students rejects wellness curriculums as “too touchy-feely” [35]. By clarifying the relationship between news monitoring and stress, we can recommend immediate, actionable changes that benefit the long-term mental health of our health professions trainees.

Currently, we are not aware of any studies that have examined COVID-related stress components across pre-clinical health disciplines or of any studies that have investigated the role of resilience or news monitoring in the COVID-related stress response. This study aimed to identify the prevalence of COVID-related stress and news monitoring and evaluate whether (1) students with higher resilience at matriculation would report lower COVID-related stress and (2) students who monitored COVID-19-related news more frequently would experience greater overall COVID-related stress.

## Methods

### Overview

First-year students at the time of matriculation (August 2019) from the University of California, Davis (UCD) School of Medicine (SOM), School of Nursing (SON), and School of Veterinary Medicine (SVM) were eligible to complete the first installment of a 4-year, longitudinal, web-based questionnaire through Qualtrics survey software (Qualtrics, Provo, UT). Eligible first-year students were identified by student emails obtained from the school administration. The SON cohort encompasses nurse practitioner and physician assistant students, collectively called advanced practice providers. Both groups take identical courses in sequence. Non-responders were given weekly email reminders to complete the survey for three consecutive weeks. Only students who consented to the initial 2019 survey were invited to complete a follow-up survey in May of 2020. Non-responders to the May 2020 invitation were again given three weekly email reminders. The study was approved by the UCD Institutional Review Board.

### Participants

Students eligible for participation in the 2019 survey (124 SOM, 87 SON, and 149 SVM) included matriculating first-year students and those deferred from previous years. Students received a small monetary gift as incentive to participate. With student consent, we linked survey responses to demographic data and academic records that each school had routinely collected. To harmonize the race and ethnicity information (which was collected differently by each school), we created a combined race and ethnicity variable, with 4 levels: “white,” “Asian,” “Hispanic,” and “other,” with “other” consisting of black, American Indian/Alaskan Native, and Middle Eastern. If students self-identified as both “white” and “black,” “Asian,” “Hispanic,” or “other,” they are included in the corresponding non-white category (see Table 1).

**Table 1:** Demographic characteristics of first-year medical students, nursing and physician assistant students, and veterinary medical students

Characteristic	SOM (*n =* 93)	SON (*n =* 41)	SVM (*n =* 67)	*P* value^a^
Age (years), mean (SD)	25.2 (2.7)	29.7 (6.3)	22.4 (2.7)	< 0.001
Gender, *n* (%)				
Female	61 (66%)	31 (76%)	59 (88%)	0.005
Male	32 (34%)	10 (24%)	8 (12%)	
Race-ethnicity^b^, *n* (%)				< 0.001
Hispanic	22 (26%)	7 (17%)	6 (9%)	
Asian	31 (36%)	12 (29%)	17 (25%)	
White	13 (15%)	17 (41%)	44 (66%)	
Other^c^	19 (22%)	5 (12%)	0 (0%)	

*SOM*, School of Medicine; *SON*, School of Nursing; *SVM*, School of Veterinary Medicine; *SD*, standard deviation

Due to rounding, percentages may not sum to 100

^a^*P* values from Kruskal-Wallis non-parametric test for age and χ^2^ tests for gender and race-ethnicity

^b^Missing values: SOM = 8

^c^Race-ethnicity category of “other” included students who self-identified as American Indian/Alaska Native (*n* = 4), African-American (*n* = 15), or Middle Eastern descent (*n* = 5)

### Study Measures

The 2019 questionnaire included 14 scales related to resilience. The 2020 follow-up questionnaire included 12 of the 2019 scales as well as additional original questions assessing individual COVID-related stressors and the frequency of seeking COVID information. The present study investigated individual COVID-related stressors and frequency of COVID news monitoring from 2020, as well as associations between demographic characteristics, resilience from 2019, and news monitoring with COVID stress.

#### COVID-19-Related Stressors

To understand situational stress, participants in the follow-up survey are asked to rate their concerns with COVID-19 on 14 items related to school, personal health, and access to resources (see Table 2). Responses consisted of the following options: “extremely,” “very,” “somewhat,” “not at all,” and “not applicable to me.” After excluding the “not applicable to me” responses, Likert frequencies were converted to linear analogue scales, with “extremely” concerned = 3, “very” concerned = 2, “somewhat” concerned = 1, and “not at all” concerned = 0. Cronbach’s alpha was 0.87, indicating excellent internal consistency. Scores from the 14 items were summed and divided by the number of items answered, giving an overall COVID-related stress score with a possible range from 0 to 3. Higher overall scores indicated higher stress levels relating to COVID-19 circumstances.

**Table 2:** COVID-19 stressors in first-year medical, nursing and physician assistant, and veterinary medical students

	SOM (*n* = 93)	SON (*n* = 41)	SVM (*n* = 67)	*P* value^a^
In the past month, in regard to COVID-19, how concerned were you about the following?				
Availability of necessities^b^, *n* (%)				0.53
Extremely concerned	7 (8%)	3 (7%)	0 (0%)	
Very concerned	9 (10%)	2 (5%)	5 (8%)	
Somewhat concerned	39 (43%)	20 (49%)	37 (56%)	
Not at all concerned	36 (40%)	16 (39%)	24 (36%)	
Communication with administration^c^, *n* (%)				0.95
Extremely concerned	12 (13%)	3 (7%)	8 (12%)	
Very concerned	12 (13%)	9 (22%)	10 (15%)	
Somewhat concerned	47 (51%)	19 (46%)	34 (52%)	
Not at all concerned	21 (23%)	10 (24%)	14 (21%)	
Curriculum schedule changes^c^, *n* (%)				0.37
Extremely concerned	37 (40%)	15 (37%)	20 (30%)	
Very concerned	35 (38%)	13 (32%)	32 (48%)	
Somewhat concerned	17 (18%)	8 (20%)	13 (20%)	
Not at all concerned	3 (3%)	5 (12%)	1 (2%)	
Social isolation^d^, *n* (%)				0.50
Extremely concerned	19 (21%)	6 (15%)	8 (12%)	
Very concerned	17 (18%)	9 (22%)	21 (31%)	
Somewhat concerned	43 (47%)	15 (37%)	27 (40%)	
Not at all concerned	13 (14%)	11 (27%)	11 (16%)	
Personal finances^d^, *n* (%)				0.33
Extremely concerned	18 (20%)	8 (20%)	6 (9%)	
Very concerned	16 (17%)	3 (7%)	13 (19%)	
Somewhat concerned	34 (37%)	20 (49%)	27 (40%)	
Not at all concerned	24 (26%)	10 (24%)	21 (31%)	
Childcare and schooling^e^, *n* (%)				0.005
Extremely concerned	0 (0%)	3 (18%)	1 (6%)	
Very concerned	0 (0%)	1 (6%)	1 (6%)	
Somewhat concerned	2 (9%)	6 (35%)	0 (0%)	
Not at all concerned	20 (91%)	7 (41%)	14 (88%)	
Medical/psychological care access^f^, *n* (%)				0.14
Extremely concerned	6 (7%)	4 (10%)	1 (2%)	
Very concerned	14 (16%)	4 (10%)	9 (14%)	
Somewhat concerned	36 (41%)	21 (53%)	24 (38%)	
Not at all concerned	31 (36%)	11 (28%)	29 (46%)	
Stress, anxiety, and depression^g^, *n* (%)				0.95
Extremely concerned	11 (12%)	8 (21%)	9 (14%)	
Very concerned	20 (22%)	5 (13%)	19 (29%)	
Somewhat concerned	47 (52%)	19 (49%)	25 (38%)	
Not at all concerned	13 (14%)	7 (18%)	13 (20%)	
Societal response to COVID^h^, *n* (%)				1.00
Extremely concerned	35 (38%)	17 (43%)	24 (36%)	
Very concerned	32 (35%)	12 (30%)	26 (39%)	
Somewhat concerned	22 (24%)	8 (20%)	15 (22%)	
Not at all concerned	3 (3%)	3 (8%)	2 (3%)	
Personal access to COVID testing^g^, *n* (%)				0.40
Extremely concerned	7 (8%)	4 (10%)	2 (3%)	
Very concerned	7 (8%)	4 (10%)	6 (9%)	
Somewhat concerned	40 (44%)	16 (41%)	28 (42%)	
Not at all concerned	37 (41%)	15 (38%)	30 (45%)	
Personal access to PPE^i^, *n* (%)				0.02
Extremely concerned	15 (18%)	10 (27%)	4 (7%)	
Very concerned	23 (28%)	13 (35%)	18 (30%)	
Somewhat concerned	29 (35%)	7 (19%)	21 (35%)	
Not at all concerned	15 (18%)	7 (19%)	17 (28%)	
Infection of family members, *n* (%)				0.50
Extremely concerned	49 (53%)	20 (49%)	27 (40%)	
Very concerned	22 (24%)	11 (27%)	23 (34%)	
Somewhat concerned	22 (24%)	10 (24%)	16 (24%)	
Not at all concerned	0 (0%)	0 (0%)	1 (1%)	
Infection of self, *n* (%)				0.54
Extremely concerned	15 (16%)	8 (20%)	6 (9%)	
Very concerned	9 (10%)	9 (22%)	12 (18%)	
Somewhat concerned	49 (53%)	14 (34%)	36 (54%)	
Not at all concerned	20 (22%)	10 (24%)	13 (19%)	
Exams^c^, *n* (%)				0.47
Extremely concerned	31 (34%)	17 (41%)	18 (27%)	
Very concerned	28 (30%)	13 (32%)	23 (35%)	
Somewhat concerned	28 (30%)	8 (20%)	22 (33%)	
Not at all concerned	5 (5%)	3 (7%)	3 (5%)	
Overall COVID stress score, mean (SD)	1.5 (0.6)	1.5 (0.6)	1.3 (0.5)	0.49

*SOM*, School of Medicine; *SON*, School of Nursing; *SVM*, School of Veterinary Medicine; *SD*, standard deviation; *PPE*, personal protective equipment

Due to rounding, percentages may not sum to 100

^a^*P* values from Kruskal-Wallis nonparametric test for overall COVID stress score and Mantel-Haenszel *χ*^2^ tests for all other variables

“Does not apply to me” or missing responses: ^b^SOM = 2 , SVM = 1; ^c^SOM = 1, SVM = 1; ^d^SOM = 1; ^e^SOM = 71, SON = 24, SVM = 51; ^f^SOM = 6, SON = 1, SVM =4; ^g^SOM = 2, SON = 2, SVM = 1; ^h^SOM = 1, SON = 1; ^i^SOM = 10, SON = 4, SVM = 7

#### Frequency of COVID News Monitoring

Using a novel Likert scale, students in the 2020 survey were asked to indicate how often they sought health information and updates pertaining to COVID through television, newspapers, news articles, social media, friends and family, or websites. Response options included “never/almost never,” “hourly or more,” “every few hours,” “daily,” “every other day,” and “weekly or less.” For analysis, participant responses were then pooled into 3 categories: “several times per day” (comprising “hourly or more” and “every few hours”), “daily,” and “every other day or less” (comprising “every other day,” “weekly or less,” and “never/almost never”).

#### Resilience

Students in the initial 2019 survey completed the Brief Resilience Scale (BRS), a 6-item validated instrument, which has been shown to reliably measure resilience as the ability to recover from stress (Cronbach’s alpha 0.80–0.91) [23]. The scale utilizes a 5-point Likert scale for statements such as “I tend to bounce back quickly after hard times.” Responses varying from 1 to 5 for all six items were added, and the sum was then divided by the total number of questions answered, giving a range from 1 to 5. Higher mean scores indicated greater resilience.

### Statistical Analysis

We used descriptive statistics to summarize demographic characteristics, individual COVID-19 stressors, frequency of COVID news monitoring, and resilience: frequencies (percentages) for categorical variables and means and standard deviations (SD) for continuous variables. Differences in characteristics among students enrolled in the three health science schools were assessed using Chi-square tests for categorical variables and Kruskal-Wallis non-parametric tests for continuous variables (since some of these variables were skewed). When examining differences in COVID-19 stressors among schools, we used Mantel-Haenszel Chi-square tests to account for the ordinality in the data [36]. Next, using general linear models, we examined associations between overall COVID-related stress scores and the following individual variables: demographic characteristics (school, age, gender, race-ethnicity), frequency of COVID news monitoring, and resilience. We first examined univariate models (including only one independent variable in the model). Then, we added all examined independent variables in a multivariable model to assess their association with the COVID-related stress score when controlling for the effects of the other variables. Since some participants were missing race-ethnicity (4%), resilience (5%), and/or frequency of news monitoring (0.5%) responses, multiple imputation was employed. We used 10 imputations to fill in missing data and ensure in-range values, so that estimates would be based on the same sample across fitted models. All analyses were conducted on each of the 10 complete data sets, and results were combined according to Rubin’s rules [37]. All analyses were implemented using SAS Version 9.4 (SAS Institute Inc., Cary, NC). All tests were two-sided, and *p* values < 0.05 were considered statistically significant.

## Results

### Demographic Characteristics

In the 2019 survey, 110 of 124 eligible students from the SOM (89%), 48 of 87 students from the SON (55%), and 108 of 149 students from the SVM (72%) provided consent and allowed the use of their demographic characteristics in the study. Across all schools, 74% (266/360) of students provided consent for the study. Two students who provided consent but did not respond to any survey items in 2019 were not invited in the 2020 survey. For the follow-up administration (spring 2020), response rates for COVID stressors are 85% (93/110) for SOM, 85% (41/48) for SON, and 63% (67/106) for SVM (Table 1). The overall response rate for COVID follow-up questions was 76% (201/264). Most of the follow-up participants are female (*n* = 151, 75%) and white (*n* = 74/193, 38%) with a mean of 25.2 years of age (Table 1).

### Individual COVID Stressors

With regard to causes of COVID-related stress, across the health profession schools, students are most likely to be “extremely” or “very” stressed about family members getting infected (*n* = 71, 76% SOM; *n* = 31, 76% SON; *n* = 50, 75% SVM), societal responses to COVID (*n* = 67, 73% SOM; *n* = 29, 73% SON; *n* = 50, 75% SVM), curriculum schedule changes (*n* = 72, 78% SOM; *n* = 28, 68% SON; *n* = 52, 79% SVM), and school examinations (*n* = 59, 64% SOM; *n* = 30, 73% SON; *n* = 41, 62% SVM) (Table 2). The overall COVID stress did not differ significantly across schools. However, SON students were more likely to be “extremely” and “very” (*n* = 23, 62%) concerned about access to personal protective equipment (PPE) compared to those in the SOM (*n* = 38, 46%) and SVM (*n* = 22, 37%) (*p* = 0.02). SON students also had greater concerns about childcare and schooling (*p* = 0.005) (Table 2).

### Associations Between Demographic Characteristics, Frequency of Monitoring COVID News, Resilience, and COVID-Related Stress Score

Female students reported greater COVID-related stress (mean = 1.47, SD = 0.54) than their male peers (mean = 1.25, SD = 0.62) and this difference remained significant in multivariable analyses (*p* = 0.045) (Table 3). Older age was associated with higher COVID stress (*p* = 0.008), while race-ethnicity was not associated with COVID stress. Among the health science schools, the SVM had lower overall COVID stress than SOM and SON, but the difference did not reach statistical significance (Table 3).

**Table 3:** Unadjusted and adjusted associations of demographic characteristics, frequency of COVID-19 news monitoring, and resilience scores with perceived overall COVID-related stress scores in first-year medical, nursing and physician assistant, and veterinary medical students

		*COVID* stress Mean (*SD*)	Unadjusted analysis^a^		Adjusted analysis^a^	
Characteristic	*n*		*Estimate* (SE)	*P* value	*Estimate* (*SE*)	*P* value
Demographic						
School				*0.41*		*0.41*
SOM	93	1.45 (0.60)	Reference	–	Reference	–
SON	67	1.46 (0.60)	0.01 (0.11)	0.89	− 0.06 (0.12)	0.61
SVM	41	1.34 (0.49)	− 0.11 (0.09)	0.24	0.11 (0.11)	0.30
Age	201	–	0.02 (0.01)	0.02	0.03 (0.01)	0.008
Gender						
Female	151	1.47 (0.54)	0.22 (0.09)	0.02	0.18 (0.09)	0.045
Male	50	1.25 (0.62)	Reference	–	Reference	–
Race-ethnicity				*0.09*		*0.18*
Hispanic	35	1.54 (0.60)	0.24 (0.12)	0.04	0.22 (0.12)	0.06
Asian	60	1.44 (0.62)	0.13 (0.10)	0.18	0.14 (0.10)	0.15
White	74	1.31 (0.50)	Reference	–	Reference	–
Other^b^	24	1.55 (0.52)	0.26 (0.13)	0.046	0.25 (0.14)	0.08
Frequency of COVID information searches				*0.009*		*0.02*
Several times per day	23	1.62 (0.56)	0.13 (0.13)	0.33	0.13 (0.12)	0.29
Daily	90	1.50 (0.58)	Reference	–	Reference	–
Every other day or less	87	1.29 (0.52)	− 0.20 (0.08)	0.02	− 0.18 (0.08)	0.03
Resilience Score	191	–	− 0.20 (0.07)	0.003	− 0.23 (0.07)	< 0.001

*SOM*, School of Medicine; *SON*, School of Nursing; *SVM*, School of Veterinary Medicine; *SD*, standard deviation; *SE*, standard error

Italic *P* values indicate significance of the overall tests for the categorical predictors with more than two levels

^a^To account for the fact that some variables were missing data, unadjusted and adjusted estimates were calculated after generating 10 complete data sets using multiple imputation, analyzing each data set, and pooling the results

^b^Race-ethnicity category of “other” included students who self-identified as American Indian/Alaska Native (*n* = 4), African-American (*n* = 15), or Middle Eastern descent (*n* = 5)

Across all schools, most students followed COVID-related news updates daily (*n* = 90, 45%) (Table 3). Mean levels of resilience were 3.59 (SD = 0.61). In the multivariable analysis, both news monitoring and resilience were independently associated with COVID stress. Greater frequency of COVID news monitoring was significantly associated with COVID-related stress (*p* = 0.02). Specifically, students monitoring the news several times per day (mean = 1.62, SD = 0.56) or daily (mean = 1.50, SD = 0.58) had significantly higher stress than those who monitored the news every other day or less (mean = 1.29, SD = 0.52). Higher resilience at matriculation is associated with lower COVID-related stress 10 months later (*p* < 0.001) (Table 3).

## Discussion

This study sought to identify COVID-related stressors in health profession students and characteristics that may mitigate or exacerbate those stressors. Although the overall degree of COVID-related stress did not differ between the three health professions schools, SON students were more troubled by the pandemic’s impact on PPE availability, childcare, and child schooling. These differences may reflect the greater proportion of SON respondents balancing roles as parents and students and the extensive patient contact that SON students experience throughout their first year. Students from all schools were remarkably concerned regarding changes in curriculum schedules and examinations, suggesting that definitive curriculum schedules and guidelines from school administrations and national health professions associations are needed to manage student stress during COVID.

While the sparse literature on COVID news monitoring has yielded conflicting associations with anxiety, our study found that excessive news monitoring behavior was associated with greater COVID-related stress [4–6]. News monitoring may help students appreciate the severity of COVID-19 and increase their sense of social responsibility, but seeking updates several times a day may incidentally generate overwhelming pressure and uncertainty [38]. Our preliminary findings on news monitoring present an avenue for further research that may address wellness criticisms of inaccessibility and sentimentality [35]. Such research should delineate motivations behind news monitoring, such as avoidance of academic responsibilities or concern on behalf of a vulnerable family member.

COVID-19 has further disrupted the developing social support networks and structured learning environment that normally bolster health science student wellbeing [18]. The cumulative stressors from this pandemic may increase the risk of rapid mental health deterioration in the later years of health professions training, which may compromise patient safety [39, 40]. With higher resilience at matriculation predicting lower overall COVID stress 10 months later, our study highlights the powerful, protective role of resilience in the setting of this pandemic. Further research should perform a cost-benefit analysis of resilience training programs, taking careful consideration of short-term academic outcomes, long-term mental health outcomes, and available school resources. Resilience training need not be extensive; rather, it can focus on providing a variety of tools and strategies that students can select based on their individual preferences and time constraints [35].

This study had several limitations commonly found in cross-sectional studies addressing rapidly evolving current events. Although our study is strengthened by including students from three different schools, they all represent only one university in California—a state with a high risk for COVID infections. Additionally, the individual COVID stressors and frequency of news monitoring questions were not validated and survey responses were collected in May 2020; therefore, responses may not reflect the current state of COVID-induced stress among students.

In summary, our study showcases resilience as a crucial protective process not only amid the daily academic rigor of health professions training but also during unprecedented crises such as COVID-19 [28]. Our findings also conceptually introduce the reduction of COVID news monitoring as an opportunity for future research. As the pandemic continues to strain academic schedules and amplify concern for family members, more longitudinal research is needed to identify factors that influence news monitoring frequency, characterize the many components of resilience, and understand changes in resilience over time. In the meantime, health professions institutions should address student concerns with curriculum disruptions and consider implementing resilience education.
